# The influence of weight on psychosocial well-being in diabetes

**DOI:** 10.1186/s40359-023-01185-4

**Published:** 2023-04-29

**Authors:** Sydney H. Telaak, Kristi A. Costabile, Susan Persky

**Affiliations:** 1 Social and Behavioral Research Branch, National Human Genome Research Institute, NIH, 31 Center Drive, B1B36, Bethesda, MD 20892 USA; 2 Department of Psychology, Iowa State University, Lagomarcino Hall, 901 Stange Road, Ames, IA 50011 USA

**Keywords:** Diabetes, Type 1 diabetes, Type 2 diabetes, Weight, Stigma, Psychosocial well-being, Health psychology

## Abstract

**Background:**

Individuals with diabetes experience a wide variety of psychosocial responses to their illness due, in part, to the nature of type 1 and type 2 diabetes. Variation in patient weight may play a central role in these differences, yet its influence on psychosocial variation is largely unknown. The current study investigates the relationship between patients’ perceived weight status and aspects of psychosocial well-being among individuals with type 1 diabetes (T1D) and type 2 diabetes (T2D).

**Methods:**

Individuals who were diagnosed with type 1 or type 2 diabetes were assessed via an online survey from the Diabetes, Identity, Attributions, and Health Study. Participants were categorized into a lower v. higher weight status group based on their self-reported perceived weight. Analyses of covariance were conducted to assess differences in measures of disease onset blame, diabetes stigma, and identity concerns among diabetes type and perceived weight status. Covariates included in our models were gender, age, education, and time since diagnosis. Bonferroni correction was used for post-hoc tests to assess any significant interactions found in our models.

**Results:**

Findings indicated that weight moderates multiple psychosocial outcomes pertinent to illness experience. Those with T2D and lower weight blamed themselves less for their disease onset, while those with higher weight felt blamed more for their disease onset by others, regardless of diabetes type. Individuals with T1D and higher weight were more frequently and more concerned about being mistaken for having the other disease type (i.e., T2D) compared to those with lower weight.

**Conclusions:**

Weight is a key influence on the psychosocial outcomes for people with diabetes, but it operates differently in type 1 versus type 2 diabetes. By further examining the unique interaction between disease type and weight status we may be able to improve psychological well-being among affected individuals of all sizes.

## Introduction

Individuals with diabetes experience varying psychosocial responses to their illness due, in part, to the nature of type 1 diabetes (T1D) and type 2 diabetes (T2D) and due to other individual differences apart from disease type. One central factor in this regard is patient weight. Common stereotypes suggest that individuals affected by T1D are likely to be lean whereas those with T2D tend to be heavy; however, weight status is not homogeneous within either type [1]. Research on diabetes and weight status has primarily focused on higher weight in relation to clinical outcomes. The present study explores the impact of perceived weight status among individuals with T1D and T2D on psychosocial outcomes such as blame, internalized stigma, and affective response to diabetes type mis-categorization to better characterize variations in illness experiences linked to weight and identify potential barriers to optimal psychosocial functioning among affected individuals.

Although approximately 70% of adults with T1D meet criteria for overweight, [2, 3] researchers have found that individuals with T1D are stereotypically viewed as “lean” while those with T2D are stereotypically viewed as “heavy” [4, 5]. This is likely due to T2D’s causal linkage and high co-occurrence with obesity, while T1D is typically associated with a strictly controlled diet. In addition, while T2D is seen as being caused largely by lifestyle factors, T1D is seen as being caused by genetics, viruses, and other uncontrollable factors unrelated to weight [6, 7].

As such, individuals with T2D are frequent targets of weight stigma and blame, and in turn experience feelings of depression, worry, anger, and overall distress [8, 9]. While these psychosocial outcomes are important on their own, these experiences can also go on to affect diabetes-related health outcomes, including poorer diabetes management and health-care disengagement [10, 11]. Psychosocial outcomes among those with T1D as a function of weight stigma are lesser studied, but existing studies report similar outcomes to those of T2D such as increased feelings of depression and distress [12].

In general, it is unknown whether differences in one’s own perceived weight influences one’s illness experience, and how this may differ by diabetes type. We focus on perceived as opposed to objective weight status (e.g., BMI) because past research has shown perceived weight to be more relevant for psychological outcomes associated with weight and weight stigma [13]. BMI is also increasingly understood to be a flawed measure, often weakly related to health risk [14]. The current analysis assesses interactions between perceived weight status and diabetes type on psychosocial factors including blame, stigma, and identity concern. These factors have important implications for psychological well-being, health management behavior, and health outcomes across the spectrum of chronic diseases and health conditions [15–19].

### Influence of weight on psychosocial outcomes in diabetes

#### Perceived blame for disease onset

Perceived blame refers to the extent to which individuals affected with diabetes perceive that they are judged by others for factors related to their disease; we focus here on disease onset. In T2D, perceived blame for disease onset is a salient aspect of the illness experience and is highly related to stigma and self-blame [17, 20]. Such blame stems from the general stereotype that T2D is a “self-inflicted condition” [7, 17, 20]. High weight status is likely to exacerbate this blame because higher weight might be considered evidence of the “bad” behavior that led to disease onset and continuation. Furthermore, T2D-affected individuals with higher weight experience both diabetes- and weight-related blame in tandem, which may be mutually reinforcing [20]. In T1D, blame tends to arise when diabetes management is perceived as poor [21, 22]. Interestingly, perceived blame related to onset has been documented as a result of T1D’s association with T2D, thought to be fueled by confusion over diabetes type or misconceptions about T1D’s controllability [17, 22].

#### Self-blame for disease onset

Self-blame can develop from internalizing judgment from others as well as individuals’ understanding of their own disease trajectory. Individuals with T2D frequently report blaming themselves for multiple aspects of their disease, including onset and suboptimal management [9, 11, 20]. Qualitative work suggests that self-blame tends to be salient to the experience of T2D, regardless of one’s weight [20].

Investigation of self-blame among individuals with T1D is sparse, but the literature does support its occurrence. A study in one young adult population with T1D found that self-blame about disease management was one of the most important predictors of psychological maladjustment [23]. Weight has not been studied as a potential factor in self-blame for diabetes onset.

#### Stigma

Diabetes stigma is frequently cited as a root cause of a variety of negative psychological outcomes among affected individuals (e.g., worry/anxiety, concealment) [17]. Attitudes driving diabetes stigma are qualitatively different between T1D and T2D, reflecting variations in disease characteristics and in relationship with weight [21, 24]. Consistent with stigma theory, variations in perceived disease controllability and stability between T1D and T2D are associated with differential stigma outcomes both in terms conceptual facets of stigma and the overall extent to which each disease is negatively viewed [25, 26]. In a qualitative study, Browne and colleagues identified stigma stemming from perceptions of T2D as a lifestyle disease associated with labels such as “fat” or “lazy” [20]. Such stereotypes were endorsed by the media, health-care professionals, family/friends, as well as affected individuals themselves, resulting in an environment of blame and judgment. Individuals with T2D often internalize these messages which causes self-stigma and feelings of shame, guilt, or embarrassment. In turn, many were unwilling to disclose their diabetes status to others for fear of being treated differently (i.e., experiences of rejection, exclusion, discrimination) in various social settings. There is some debate as to how this relates to weight status; several research participants posited that obesity was the root cause of this scrutiny, implying that T2D stigma may be a form of weight stigma.

T1D-specific stigma also involves similar components of judgment and discrimination. Again, blame and judgment can arise from perceived irresponsibility in diabetes management. A salient component of T1D stigma is misplaced judgment, or “stigma by association” with T2D [22]. Like those with T2D, those with T1D report feelings of self-consciousness and differential treatment in social situations as a result of these assumptions. In relation to weight, one study found no effect of weight on consequences of diabetes-related stigma for social and emotional outcomes among individuals with T1D [27]. It is plausible that T1D stigma and weight may be related in several ways. First, high weight status may be perceived as an indicator for suboptimal diabetes management, resulting in increased stigmatization by way of blame and judgment. Second, high weight status may increase misperception of diabetes type (i.e., being wrongly perceived as having T2D), resulting in identity-related stigmatization.

#### Diabetes mis-categorization

Diabetes mis-categorization, or being mistaken for having the incorrect diabetes type, has been recognized as a salient factor of T1D illness experience [22, 28]. Qualitative literature suggests that, among those with T1D, fears of being mistaken as having T2D are common and often coincide with having higher weight status [22, 28]. Concern about mis-categorization has been linked to adverse psychological consequences such as feelings of self-consciousness and anxiety, but this has not been assessed quantitatively [28].

There is no literature to date on the existence or psychological impact of diabetes mis-categorization for those with T2D. Mis-categorization may be more likely if an individual with T2D does not fit into the stereotype of being “heavy.” Preferred self-categorization by affected individuals suggests that T1D is perceived as the “higher status subgroup.” [29]. Some data suggests that T1D is perceived to be less stigmatized than T2D by those affected with T2D, [20] however affected individuals with T1D tend to perceive more stigmatization [27]. Regardless, the dimensions on which T1D and T2D are stigmatized vary [21, 24]. Mis-categorization of individuals with T2D may be relatively socially beneficial in that it relieves individuals of stigmatizing aspects of their own illness identity.

### Current study

**Fig. 1:**
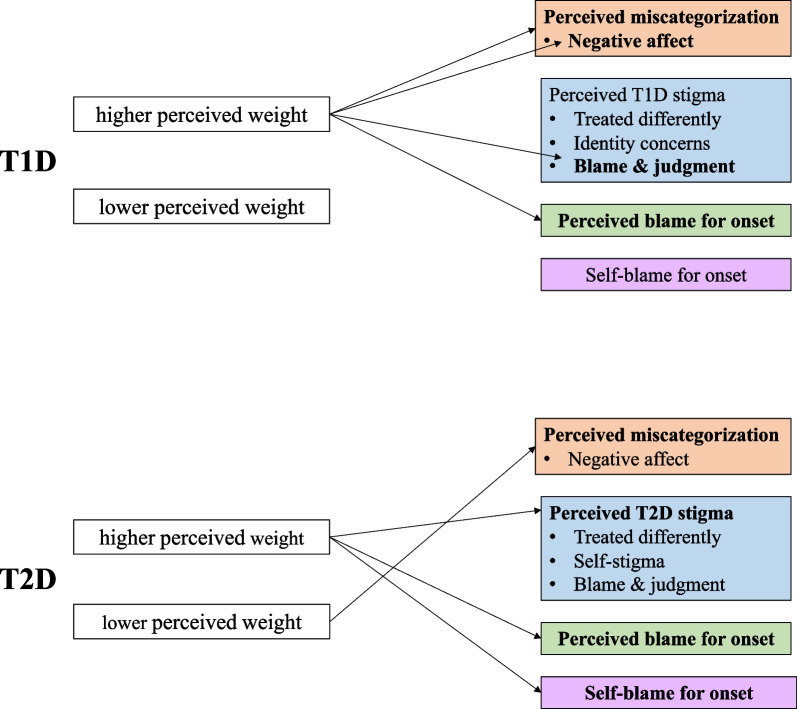
Framework of hypotheses. Arrows denote that perceived weight status group is associated with higher levels of relevant outcome

The current study investigates perceived blame and self-blame for disease onset, diabetes stigma, and negative affect in response to diabetes mis-categorization in relation to perceived weight status in a sample of adults affected by T1D or T2D (see Fig. 1).

We hypothesized the following:

1. Among those with T2D, individuals with a lower weight status will report lower levels of self-blame for their disease onset, and those with a higher weight status will report higher levels of self-blame for their disease onset. Self-blame will not differ by weight status for those with T1D.
  1. Perceived blame will differ by weight status such that those with higher weight status will perceive more blame across both diabetes types.
2. Individuals with T2D and a higher weight status will report higher levels on all facets of diabetes stigma than those with a lower weight status. For participants with T1D, individuals with a higher weight status will report higher levels of stigma on the blame and judgment stigma subscale than those with a lower weight status. Due to the qualitative differences in diabetes stigma between T1D and T2D, these facets of stigma cannot be directly compared across disease type.
3. Individuals who are incongruent with their stereotype (i.e., T1D/higher weight status and T2D/lower weight status) will more frequently be mis-categorized compared to those who are congruent with their stereotype.
  3a Those with T1D and a higher weight status will report higher levels of negative affect about mis-categorization.

## Methods

### Participants

Data came from the Diabetes, Identity, Attributions, and Health Study (DINAH), a cross-sectional study conducted in 2017 which investigates causal attributions for diabetes in relation to affected and unaffected individuals’ perceptions, management, and attitudes surrounding diabetes [6]. Eligible participants for this analysis included adults who self-reported their diagnosis by a health care professional with either T1D or T2D. Diagnosed individuals were recruited through ResearchMatch (a non-for-profit online registry for clinical studies) and Facebook (advertised through established diabetes groups). Participants diagnosed with T1D or T2D were compensated $10.00 upon completion of the survey. For data quality purposes, participants were required to demonstrate basic knowledge about diabetes and were asked at the end of the survey to re-confirm their diabetes status; those who did not meet these data quality expectations were compensated but excluded from the final dataset (n = 27). Procedures for coding diabetes knowledge and screening are available elsewhere; [6] briefly, participants who were not able to correctly identify at least two dimensions on which T1D and T2D differed were judged to have poor knowledge and were excluded from analysis. The sample for this analysis consisted of 372 participants diagnosed with either T1D (n = 182) or T2D (n = 190). This study was not preregistered.

### Measures

#### Perceived weight status

Participants reported their perceived weight status with a single item (i.e., right now, do you think you are...?). This was measured on a 4-point scale (1 = underweight, 2 = about right, 3 = overweight, 4 = very overweight).

#### Perceived blame

Participants reported on perceived blame with a single item (i.e., “I feel that other people think I am to blame for my diabetes”). This was measured on a 4-point scale (1 = strongly disagree, 4 = strongly agree). This measure was created a priori as a face-valid assessment.

#### Self-blame

Participants reported on self-blame with a single item (i.e., “I feel I am to blame for my diabetes”). This was measured on a 4-point scale (1 = strongly disagree, 4 = strongly agree). This measure was created a priori as a face-valid assessment.

#### T1D stigma

Participants with T1D reported on stigma with the Diabetes Stigma Assessment Scale (DSAS-1), a 19-item scale developed to assess perceived and experienced stigma specific to adults with T1D. [21] This scale assesses T1D stigma through three subscales: Treated Differently (6 items, Cronbach’s α = 0.87), Identity Concerns (7 items, Cronbach’s α = 0.89), and Blame/Judgment (6 items, Cronbach’s α = 0.86). Each item was measured on a 5-point scale (1 = strongly disagree, 5 = strongly agree).

#### T2D Stigma

Participants with T2D reported on stigma with the Diabetes Stigma Assessment Scale (DSAS-2), a 19-item scale developed to assess perceived and experienced stigma specific to adults with T2D [24]. This scale assesses T2D stigma through three subscales: Treated Differently (6 items, Cronbach’s α = 0.89), Self-Stigma (7 items, Cronbach’s α = 0.92), and Blame/Judgment (6 items, Cronbach’s α = 0.89). Each item was measured on a 5-point scale (1 = strongly disagree, 5 = strongly agree).

#### Diabetes Mis-categorization: frequency

Participants reported on diabetes mis-categorization with a single item to assess the extent to which they are mis-categorized by other people as having the incorrect diabetes type (i.e., “People mistakenly believe that I am affected by T2D (T1D survey version)/T1D (T2D survey version)). This was measured on a 5-point scale (1 = never, 5 = always).

#### Diabetes Mis-categorization: negative affect

Participants reported on diabetes mis-categorization with a single item to assess the extent to which they would feel upset if mis-categorized as having the wrong diabetes type by another person (i.e., “It would upset me if someone mistakenly believed I was affected by T2D (T1D survey version)/T1D (T2D survey version)). This was measured on a 7-point scale (1 = strongly disagree, 7 = strongly agree).

### Procedure

Participants completed this online survey anonymously via SurveyMonkey. After consenting to the study through an online consent form, individuals responded to a variety of items including self-blame for disease onset, perceived blame for disease onset, diabetes stigma, items related to diabetes mis-categorization, demographics (e.g., gender, race/ethnicity, age, time since diagnosis, etc.), and perceived weight status. Measures were administered to all participants except for the Diabetes Stigma Assessment Scales, which were specific to diabetes type.

### Analysis

Perceived weight status was coded as a binary variable due to the categorical nature of this item and its non-normal distribution. Participants who responded “underweight” or “about right” for this item were combined into a group labeled “lower perceived weight status” (n = 114), and respondents who responded “overweight” or “very overweight” for this item were combined into a group labeled “higher perceived weight status” (n = 258). A binary categorization was chosen to ensure large enough group sizes for analysis.

Analyses of covariance (ANCOVA) were conducted using a univariate general linear model to assess differences in measures by diabetes type and perceived weight status. Covariates included in our models were gender, age, education, and time since diagnosis, where there was difference between demographic groups and theoretical reason to believe a given variable may be a confounder. Bonferroni correction was applied to post-hoc tests assessing significant interactions.

## Results

Sample characteristics and descriptive statistics for the sample can be found in Table 1.

**Table 1:** Sample characteristics and descriptive statistics for affected individuals included in analyses

	Lower perceived weight status (n = 114)	Higher perceived weight status (n = 258)	Total (n = 372)
**T1D (n = 182)**	**75**	**107**	**182**
Gender—woman (%)*	51 (68%)	87 (82.1%)	138 (76.2%)
Race—white (%)	68 (91.9%)	92 (87.6%)	160 (89.4%)
Ethnicity—non-Hispanic (%)	73 (97.3%)	101 (95.3%)	174 (96.1%)
Education—college graduate (%)	56 (74.4%)	70 (65.4%)	126 (69.2%)
Age (mean)	39.95 (14.97)	40.70 (13.88)	40.39 (14.30)
Time since Diagnosis (mean)	21.86 (16.44)	23.22 (13.79)	22.67 (14.90)
**T2D (n = 190)**	**39**	**151**	**190**
Gender—female (%)***	17 (43.6%)	118 (78.1%)	135 (71.1%)
Race—white (%)	28 (73.7%)	121 (81.2%)	149 (79.7%)
Ethnicity—non-Hispanic (%)	36 (92.3%)	147 (97.4%)	183 (96.3%)
Education—college graduate (%)	24 (61.5%)	78 (51.7%)	102 (53.7%)
Age (mean)	54.49 (12.07)	53.89 (11.35)	54.01 (11.47)
Time since Diagnosis (mean)	7.923 (10.05)	10.40 (9.11)	9.89 (9.34)
**Total (n = 372)**	**114**	**258**	**372**
Gender—woman (%)***	68 (59.6%)	205 (79.8%)	273 (73.6%)
Race—white (%)	96 (85.7%)	213 (83.9%)	309 (84.4%)
Ethnicity—non-Hispanic (%)	109 (95.6%)	248 (96.5%)	357 (96.2%)
Education—college graduate (%)*	80 (70.2%)	148 (57.4%)	228 (61.3%)
Age (mean)*	44.92 (15.61)	48.42 (14.04)	47.35 (14.61)
Time since Diagnosis (mean)	17.05 (15.96)	15.76 (12.94)	16.16 (13.92)

	T1D (n = 182)	T2D (n = 190)	*P* value
Gender—woman (%)	138 (76.2%)	135 (71.1%)	0.257
Race—white (%)	160 (89.4%)	149 (79.7%)	0.010*
Ethnicity—non-Hispanic (%)	174 (96.1%)	183 (96.3%)	0.926
Education—college grad (%)	126 (69.2%)	102 (53.7%)	0.002**
Age (mean)	40.39 (14.3)	54.01 (11.47)	0.000***
Time since diagnosis (mean)	22.67 (14.9)	9.89 (9.34)	0.000***

Frequency (%) or M (SD) reported *p < 0.05 **p < 0.01 ***p < 0.001

Asterisks denote significant group differences between perceived weight status group

### Perceived blame for disease onset

See Table 2 for a summary of the means and standard deviations. There was a main effect of perceived weight status, such that there were significantly higher levels of perceived blame among those with a higher weight status compared to those with a lower weight status, F(1, 366) = 5.81, *p* = 0.016, η_p_^2^ = 0.04. There was no main effect for diabetes type and no significant interaction observed (η_p_^2^ = 0.01, 0.00, respectively).

**Table 2:** Summary of significant pairwise comparisons by diabetes type and perceived weight status

Variable	Pairwise comparisons		Mean difference	SE	*p*
	Sample subgroup	Comparison			
Self-blame	T1D	Lower v. Higher Perceived Weight	− 0.03	0.13	0.786
	T2D	Lower v. Higher Perceived Weight	− 0.45	0.15	0.003
	Lower Perceived Weight	T1D v. T2D	− 0.84	0.19	0.000
	Higher Perceived Weight	T1D v. T2D	− 1.25	0.14	0.000
Diabetes Mis-categorization: Frequency	T1D	Lower v. Higher Perceived Weight	− 0.39	0.16	0.017
	T2D	Lower v. Higher Perceived Weight	0.48	0.19	0.015
	Lower Perceived Weight	T1D v. T2D	0.67	0.25	0.007
	Higher Perceived Weight	T1D V. T2D	1.54	0.18	0.000
Diabetes Mis-categorization: Concern	T1D	Lower v. Higher Perceived Weight	− 0.65	0.27	0.016
	T2D	Lower v. Higher Perceived Weight	0.59	0.32	0.071
	Lower Perceived Weight	T1D v. T2D	1.19	0.41	0.004
	Higher Perceived Weight	T1D V. T2D	2.24	0.30	0.000

### Self-blame for disease onset

**Fig. 2:**
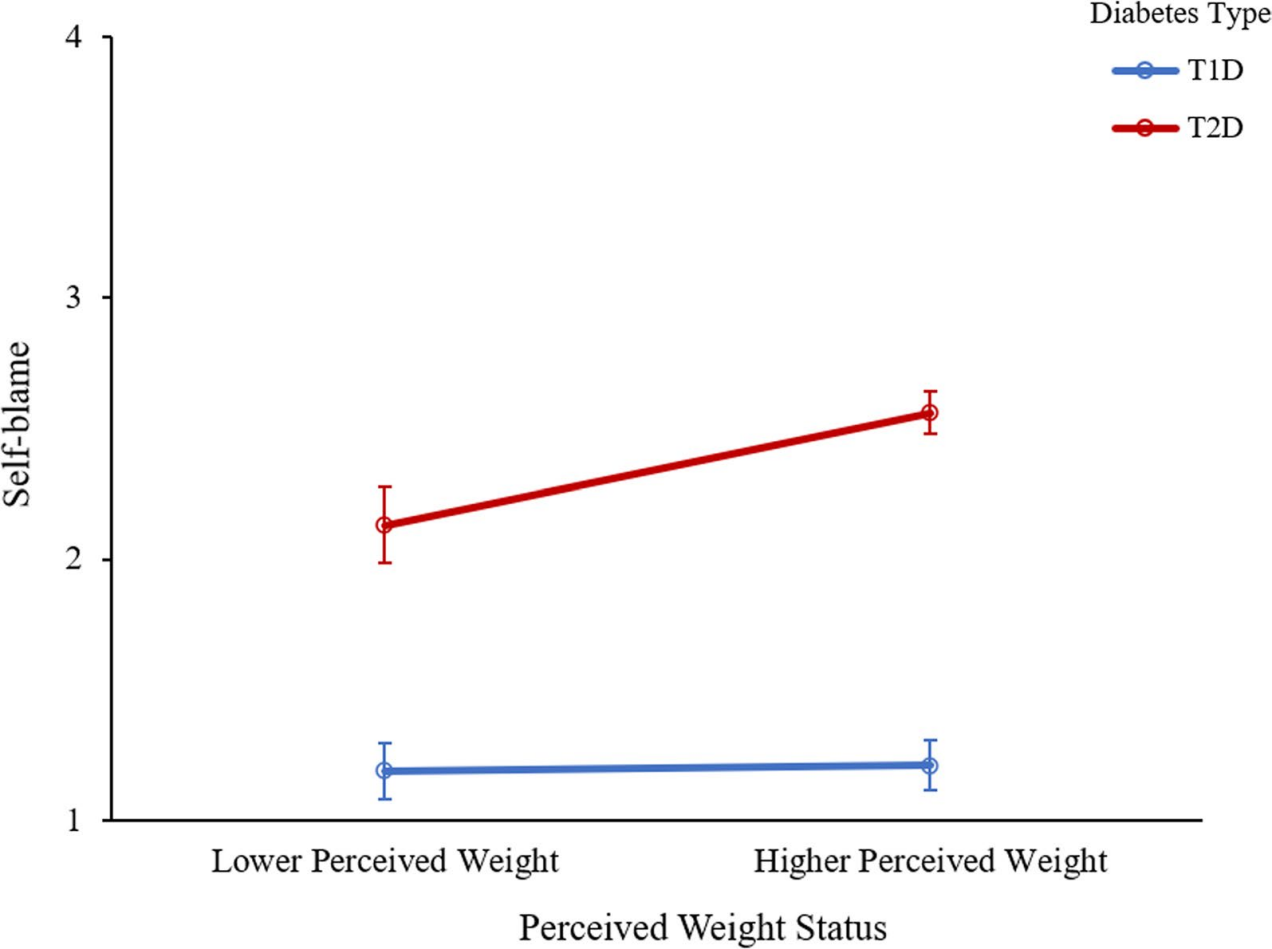
Feelings of self-blame by diabetes type and perceived weight status. Error bars represent standard error

There was a main effect of diabetes type, such that there were significantly higher levels of self-blame among those with T2D compared to those with T1D, F(1, 365) = 57.73, *p* < 0.001, η_p_^2^ = 0.14. There was also a main effect of perceived weight status, such that there were significantly higher levels of self-blame among those with a higher weight status compared to those with a lower weight status, F(1, 365) = 57.73, *p* = 0.015, η_p_^2^ = 0.02. Main effects were qualified by a significant interaction between diabetes type and perceived weight status, F(1, 365) = 4.67, *p* = 0.031, η_p_^2^ = 0.01 (See Fig. 2). Specifically, among participants with T2D, those with a lower perceived weight status reported significantly lower levels of self-blame than those with a higher perceived weight status (see Table 3 for pairwise comparisons). Participants with T1D did not differ in their self-blame as a function of their perceived weight status.

**Table 3:** Summary of means and standard deviations by diabetes type and perceived weight status

Variable	Diabetes Type	Perceived Weight Status			
		Lower Perceived Weight Status		Higher Perceived Weight Status	
		M	SD	M	SD
Perceived Blame	T1D	2.03	0.99	2.29	1.04
	T2D	1.95	1.12	2.42	1.15
Self-Blame	T1D	1.19	0.54	1.21	0.52
	T2D	2.13	0.95	2.56	1.04
DSAS-1	T1D	52.93	15.64	56.78	15.48
Blame and Judgment		2.30	0.95	2.34	0.99
Identity Concerns		2.55	1.06	2.83	1.07
Treated Differently		3.56	0.97	3.86	0.80
DSAS-2	T2D	42.70	19.28	49.36	18.22
Blame and Judgment		2.65	1.09	3.16	1.12
Treated Differently		2.01	1.15	2.09	0.95
Self-stigma		2.07	1.13	2.40	1.18
Diabetes Mis-categorization: Frequency	T1D	3.03	1.16	3.42	1.10
	T2D	2.00	1.38	1.60	0.92
Diabetes Mis-categorization: Concern	T1D	4.20	2.13	4.88	1.81
	T2D	2.44	1.88	1.97	1.49

### Stigma

#### T1D stigma

**Fig. 3:**
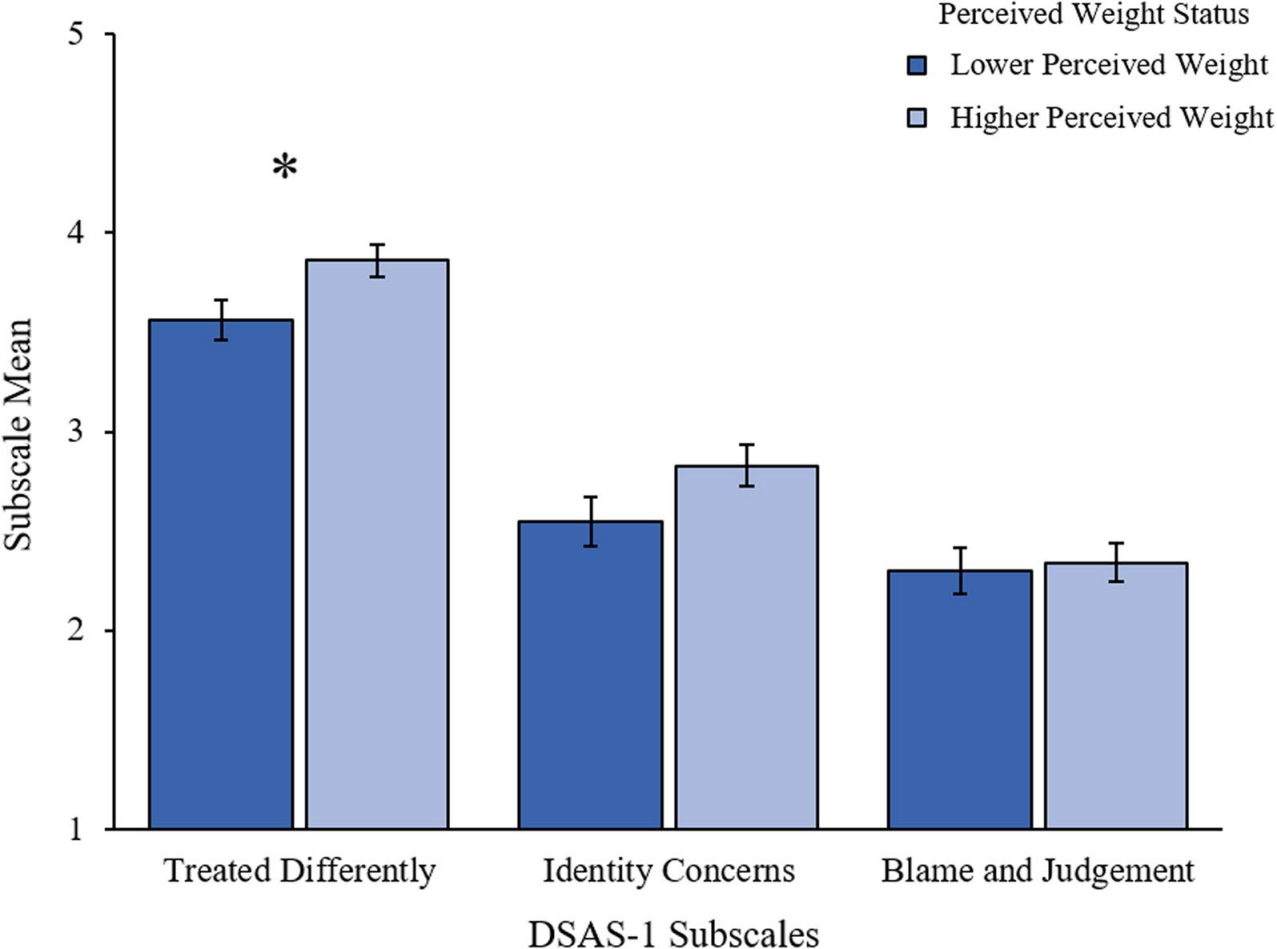
Type 1 Diabetes Stigma Assessment Scale (DSAS-1) subscales by perceived weight status. Error bars represent standard error

A main effect of perceived weight status was observed on the Treated Differently Subscale, such that there was a significantly higher level of perceived negative differential treatment among those with a higher weight status compared to those with a lower weight status, F(1, 178) = 4.50, *p* = 0.035, η_p_^2^ = 0.03 (See Fig. 3). A similar pattern was also observed for the Identity Concerns Subscale although this did not reach significance, F(1, 178) = 3.87, *p* = 0.051, η_p_^2^ = 0.02. No significant differences were found for the Blame/Judgment Subscale, η_p_^2^ = 0.00.

#### T2D stigma

**Fig. 4:**
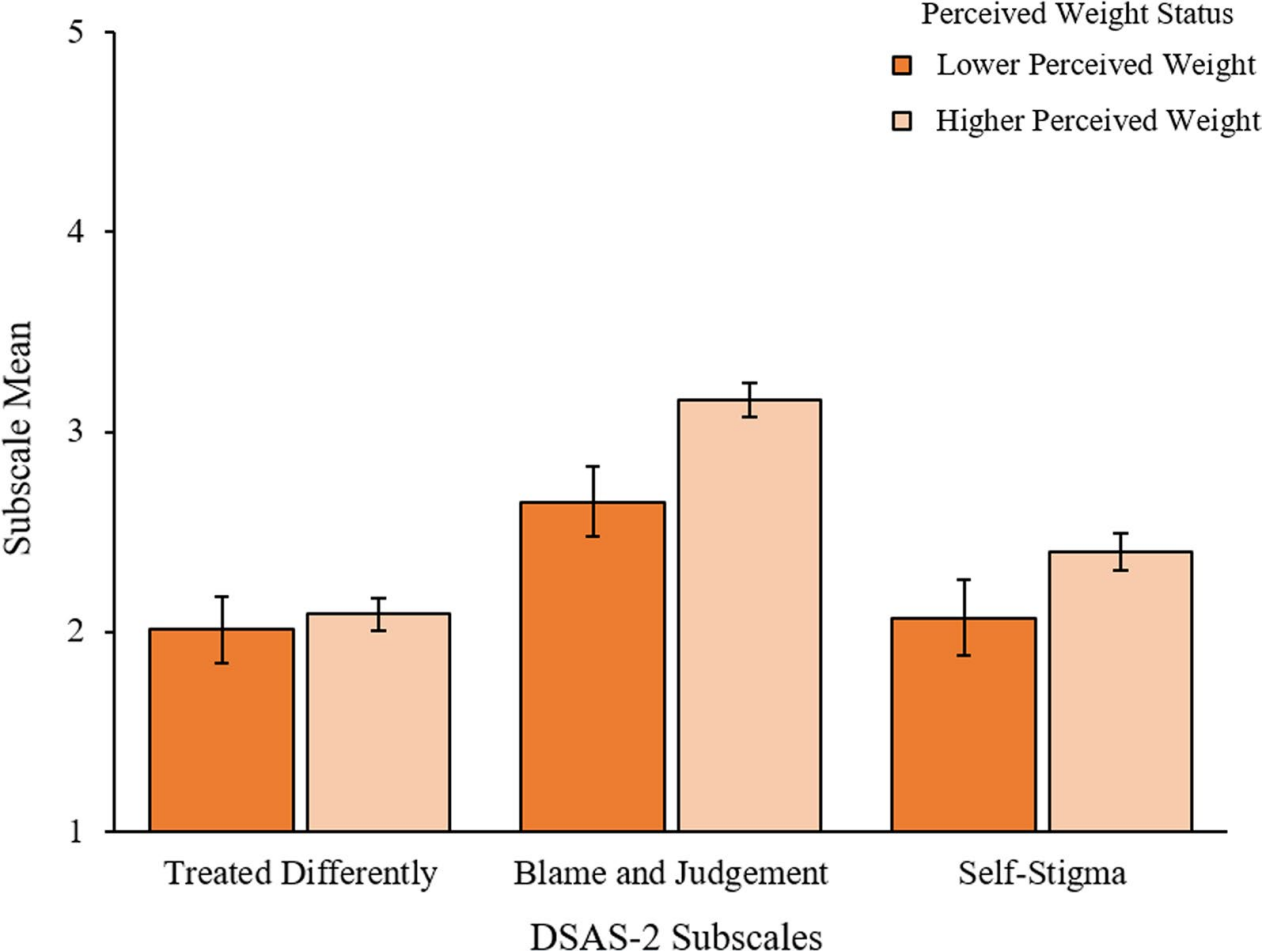
Type 2 Diabetes Stigma Assessment Scale (DSAS-2) subscales by perceived weight status. Error bars represent SE

There were no significant differences by perceived weight status on any of the subscales (Treated Differently, Blame/Judgement, and Self-stigma η_p_^2^ = 0.00, 0.02, and 0.01, respectively) (See Fig. 4).

### Diabetes mis-categorization

#### Diabetes mis-categorization: frequency

**Fig. 5:**
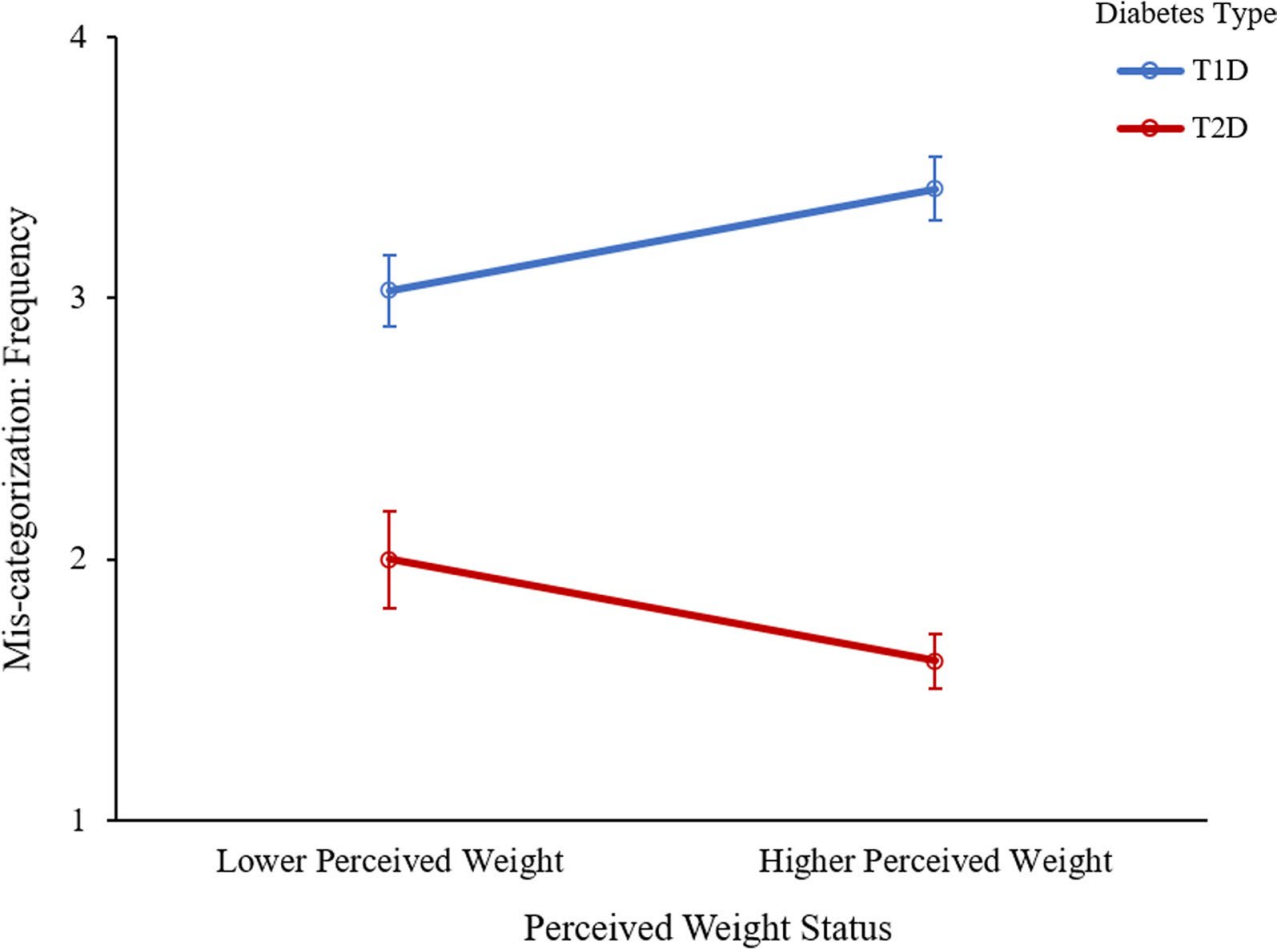
Frequency of diabetes mis-categorization by diabetes type and perceived weight status. Error bars represent standard error

There was a main effect of diabetes type, such that there were significantly higher levels of diabetes mis-categorization among those with T1D compared to those with T2D, *F*(1, 363) = 38.99, *p* < 0.001, η_p_^2^ = 0.09 (see Fig. 5). A significant interaction was found between diabetes type and perceived weight status, *F*(1, 363) = 12.07, *p* = 0.001, η_p_^2^ = 0.03. Specifically, among those with T1D, those with a higher weight status reported diabetes mis-categorization more frequently than those with a lower weight status. Among those with T2D, this relationship was in the opposite direction, such that those with a lower weight status reported diabetes mis-categorization more frequently than those with a higher weight status.

#### Diabetes mis-categorization: negative affect

**Fig. 6:**
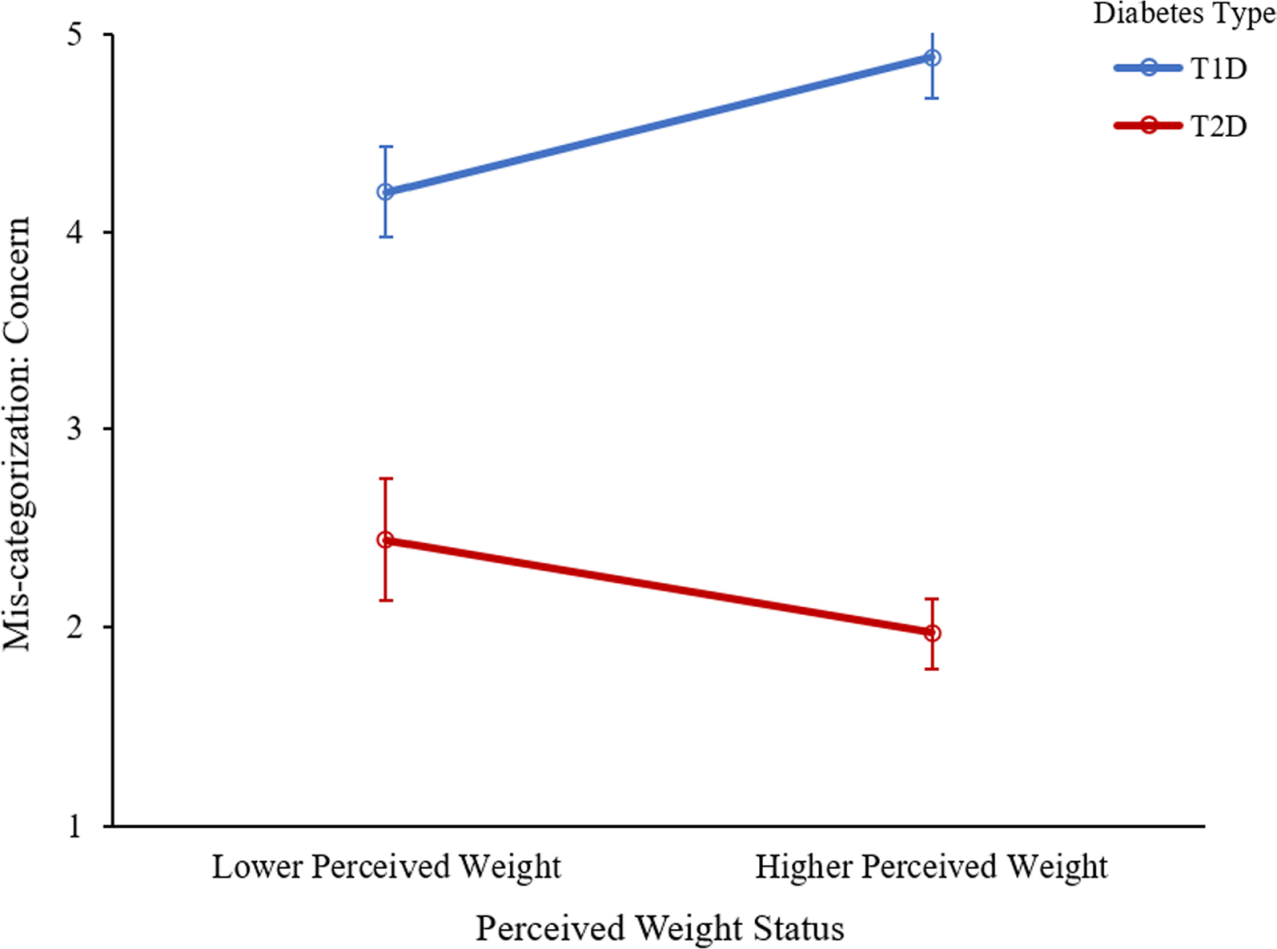
Upset over mis-categorization by diabetes type and perceived weight status. Error bars represent standard error

There was a main effect of diabetes type, such that there were significantly higher levels of feeling upset over mis-categorization among those with T1D compared to those with T2D, *F*(1, 366) = 34.48, *p* < 0.001, η_p_^2^ = 0.09 (See Fig. 6). A significant interaction was found between diabetes type and perceived weight status, *F*(1, 366) = 8.86, *p* = 0.003, η_p_^2^ = 0.02. Specifically, among those with T1D, participants with a lower weight status reported significantly lower levels of feeling upset over diabetes mis-categorization than those with a higher weight status. Among those with T2D, this pattern trends in the opposite direction, although the relationship did not reach statistical significance (see Table 2).

## Discussion

The present study investigated the relationship between perceived weight status and several psychosocial constructs pertinent to individuals affected by diabetes that also have important influences on downstream health outcomes. Results indicated that weight is salient to the illness experience of people with both types of diabetes but operates differently between them. For individuals with T2D, lower weight acts as a buffer for a variety of internal and external negative outcomes. For those with T1D, higher weight is largely relevant for outcomes related to perceptions of how others view them.

Results were supportive of hypothesis 1, indicating that those with T2D not only exhibit higher rates of self-blame for disease onset than those with T1D, but that levels of self-blame for individuals with T2D are contingent on one’s weight while those for T1D are not. This is likely rooted in causal attributions wherein weight is tightly tied to T2D onset but is less relevant for the onset of T1D. This stands in contrast to findings on perceived blame for disease onset wherein higher weight status was associated with higher blame across diabetes types. Therefore, it is plausible that self-blame operates through causal perceptions related to disease onset, while perceived external blame for those with T1D may instead relate to perceived mis-categorization and blame that can accompany it.

Surprisingly, the extent to which affected individuals experience stigma specific to their diabetes type was largely unrelated to their perceived weight status. The Diabetes Stigma Assessment Scales were developed individually and have various similarities and differences (see Browne et al., 2017 for an in-depth discussion) [21]. Among our T1D sample, the “Treated Differently” subscale was the only aspect of stigma that was higher in relation to higher perceived weight status. This subscale encompasses experiences of exclusion or rejection in social situations, suggesting that these experiences might also be affected by weight-related stigma. Among the T2D sample, none of the stigma elements differed by perceived weight status. The Type 2 Diabetes Stigma Assessment Scale was partially based on qualitative data from affected individuals, which included discussions about weight stigma [20]. Close examination of the scales, however, indicates only one item in the Type 2 Diabetes Stigma Assessment Scale that explicitly addresses weight concerns, and participant weight was not explicitly considered in construction of the scale [20]. Consequently, this scale may capture what is unique to diabetes stigma that is non-overlapping with weight stigma. Research findings indicate the Type 2 Diabetes Stigma Assessment Scale and a weight stigma scale are only moderately correlated, [30] and that facets of diabetes stigma captured by the Diabetes Stigma Assessment Scales and weight stigma are therefore different constructs. This is yet to be studied for T1D.

In accordance with our hypotheses, individuals who were weight-incongruent with their illness stereotype (i.e., T1D/higher weight and T2D/lower weight) perceived that they were more frequently mis-categorized as the other diabetes type compared to those who were weight-congruent. Additionally, individuals with T1D were more upset about being mis-categorized than were individuals with T2D, particularly when those with T1D had higher weight. This is consistent with studies indicating that diabetes mis-categorization is a common concern among those with T1D [22, 28]. However, the current report is the first to tie this concern to weight status of individuals with T1D. This is also the first study to report the occurrence of diabetes mis-categorization among individuals with T2D.

Negative affect about mis-categorization likely stems from similar mechanisms seen in the blame-related constructs. Individuals with T1D who have higher weight may experience blame and stigma typically reserved for individuals with T2D, in that it is associated with perceptions of causal responsibility. In turn, these individuals may feel that negative perceptions and treatment are unfair or unwarranted. Individuals with T2D were less upset about mis-categorization and this was unrelated to perceived weight. Indeed, individuals with T2D perceive those with T1D as being judged less harshly for their condition, [6, 20, 29] and so diabetes mis-categorization for individuals with T2D does not have the same negative connotations. The implications of miscategorization on affected individuals may also depend on *who* is doing the miscategorizing. For example, healthcare professionals who do not fully understand the difference between T1D and T2D or incorrectly assume a patient’s diabetes type can impede the development of a proper care plan or place strain on the patient-provider relationship.

This report is the first to directly examine weight as a moderator of T2D *and* T1D illness experiences and to directly compare the two. Overall, weight status was found to be linked to experiences of self-blame, perceived blame, and responses to diabetes mis-categorization, while stigma as measured by the Diabetes Stigma Assessment Scales seems to function independently of weight status. Results suggest that, among those with T2D, lower weight acts as a protective factor against several negative psychosocial outcomes. Additionally, results for those with T1D suggest that public perceptions related to T2D and weight may have consequences for the psychosocial well-being of this community. These effects may be particularly prevalent given high rates of overweight and obesity among Americans with T1D.

Research on weight-related blame and stigma has been typically confined to understanding the experiences of those with T2D; however, the present study highlights the importance of investigating weight in the context of T1D as well. This report also sheds light on an important yet under-researched problem among the diabetes community: negative perceptions of the other diabetes type may fuel feelings of blame and concern over diabetes mis-categorization. The current results indicate that public misconceptions about weight and its relation to diabetes type can lead to a variety of psychosocial consequences that impact both the T1D and T2D communities.

## Limitations

Limitations include but are not restricted to the following. Recruitment via an online research registry and diabetes support groups likely biased the sample towards individuals who perceive diabetes as more central to their identity and may not be representative across individuals with diabetes. Participants were majority white and women, and we were unable to examine the impact of race and ethnicity in this analysis. Given racial health disparities in diabetes and that diabetes and weight-related perceptions and outcomes vary by race and ethnicity, [31–33] future research should prioritize sample diversity. We also combined participants who reported perceiving that they were “overweight” and “very overweight” in the same category although weight status may influence stigma and blame-related experiences. In terms of our measures, perceived weight was categorical by nature and was not uniformly distributed within the T2D sample, which may have impacted the sensitivity of our analyses. Additionally, our measures of blame were centered on disease onset, but did not consider other aspects of blame pertinent to diabetes experiences, such as management. The subscales “Blame and Judgment” in both DSAS scales encompass aspects of blame for suboptimal diabetes management; however, we are unable to directly compare by diabetes type due to their qualitative differences.

## Conclusion

These psychosocial themes of blame, stigma, and identity concern are known to influence diabetes self-management and risks of illness complications [10, 25]. The current results suggest that interventions related to psychological health in the context of diabetes should consider, and possibly be tailored, to address weight stigma concerns unique to each diabetes type. Interventions and discussions to mitigate such stigma have been ongoing, citing the need for education and media to focus on correcting misconceptions about what causes diabetes and obesity, and how the two relate to each other [30, 34, 35]. Going forward, research on this disease should mobilize efforts towards stigma reduction, especially for subgroups where it may be most prevalent and most detrimental. Such work has the potential to prioritize the well-being of affected individuals in an inclusive manner and take into account patients of all sizes.

## Data Availability

This study was not preregistered. We have control of primary data and agree to allow the journal to review any and all data material related to this submission. The datasets used and analyzed during the current study are available from the corresponding author on reasonable request.
